# Editorial: Biomarkers to Enable Therapeutics Development in Neurodevelopmental Disorders

**DOI:** 10.3389/fnint.2020.616641

**Published:** 2020-11-12

**Authors:** Mustafa Sahin, John A. Sweeney, Stephanie R. Jones

**Affiliations:** ^1^Boston Children's Hospital and Harvard Medical School, Boston, MA, United States; ^2^Harvard Medical School, Boston, MA, United States; ^3^Department of Radiology, Sichuan University, Chengdu, China; ^4^Department of Psychiatry, University of Cincinnati, Cincinnati, OH, United States; ^5^Department of Neuroscience, Brown University, Providence, RI, United States; ^6^Center for Neurorestoration and Neurotechnology, Providence VA Medical Center, Providence, RI, United States

**Keywords:** autism, neurodevelopmental disorders, biomarkers, EEG, MRI, MEG

Investigations over the last two decades have established that there are a large of number genes implicated in the pathogenesis of neurodevelopmental disorders such as autism. Understanding the various genetic etiologies and their phenotypic consequences have brought us to an inflection point in terms of bringing new diagnostics and, more importantly, new therapeutics for individuals affected with neurodevelopmental disorders. Due to the advances in genetics, neurobiology and computational techniques, it will soon be possible to conduct successful clinical treatment trials with mechanism-based therapies to such disorders. Given the heterogeneity of causes of neurodevelopmental disorders, the starting point for these clinical trials are rare genetic diseases. There are numerous efforts to develop small molecules and gene therapies for various neurogenetic disorders. However, the efforts to identify disease modifying treatments for neurodevelopmental disorders to date have been hampered by lack of objective and sensitive biological outcome measures. A crucial key to overcome this obstacle is the development of translational and quantative biomarkers to bolster outcomes measures in the development therapeutics of neurodevelopmental disorders.

There are many categories and potential uses of biomarkers as defined by the FDA's BEST (biomarkers, endpoints and other tools) Resource (http://www.ncbi.nlm.nih.gov/books/NBK326791/). For neurodevelopmental disorders, biomarkers that reflect molecular target engagement, pharmacodynamic response, and treatment response may provide earlier indicators of efficacy than traditional endpoints (which may take months to years). Furthermore, biomarkers could help stratify trial participants and thus reduce heterogeneity or enrich a population for maximal treatment response in early clinical trials. This Frontiers Research Topics brings together a set of articles, which investigate and/review development, validation, and use of various potential biomarkers in neurodevelopmental disorders.

Fluid biomarkers have traditionally been the easiest to assess since blood is not difficult to obtain and measurements can be quantitative. Some examples include C-reactive protein as a measure of inflammation or serum creatinine as a measure of renal function. However, whether biomarkers in blood or serum can be identified that are correlated with symptoms and pathophysiological mechanisms of neurodevelopmental disorders is not yet clear. Developing and validating these tools is thus a primary focus of neurodevelopmental disorder research. The Frontiers set of papers was organized to present an overview of current progress in this area.

Neul et al. used a non-targeted metabolomic approach to study plasma metabolite profiles from individuals with Rett Syndrome (RTT) compared to unaffected age- and gender-matched siblings. They identified significant alterations in metabolites related to oxidative stress, mitochondrial dysfunction, and alterations in gut microflora. Faundez et al. provide a broader review of the potential use of “omics” platforms to study individuals with RTT and argue that RTT is an ideal disorder to investigate molecular biomarkers due to its origin in transcriptional dysregulation. McLane et al. studied a different syndromic form of intellectual disability, Fragile X Syndrome (FXS), and present preliminary data that plasma amyloid-beta precursor protein (APP) may be dysregulated in individuals with FXS. It will be interesting to see if these alterations reported in RTT and FXS can be validated in larger and independent samples.

One possibility is that peripheral serum and blood biomarkers may not reflect the pathobiology within the central nervous system, (CNS), especially in neurodevelopmental disorders where there may not be significant ongoing neuronal damage. In this case, biomarkers directly related to CNS biochemistry and metabolism may be helpful. Alzheimer's disease is one example where biomarkers, such as PET imaging and cerebro-spinal fluid (CSF) assays, are established as sufficiently predictive of disease pathology and are being used in clinical trials. However, CSF is rarely obtained in children with neurodevelopmental disorders, and PET scanning is rarely performed unless there is a clinical indication such as epilepsy surgery workup. Therefore, we need biomarkers that are easier to obtain in this population of patients. Bridgemohan et al. asked whether it is feasible to integrate the collection of biochemical (blood serotonin, urine melatonin sulfate excretion) and clinical (head circumference, dysmorphology exam, digit ratio, cognitive, and behavioral function) biomarkers during routine ASD clinic visits. Their pilot study, which was performed in the clinical setting across multiple institutions, provides proof of feasibility for use of biomarkers that could be measured during clinical care.

While often not obtained with a clinical visit, brain imaging is widely used in neurological care. There is a rich literature of imaging studies in neurodevelopmental disorders such as autism. However, it is unclear whether structural MRI features such as volume of a specific region or thickness of the cortex will predict or reflect treatment efficacy in therapeutic intervention trials. There are rare examples of white matter pathology that seems to respond to small molecule therapies in disorders such as tuberous sclerosis complex (TSC) (Tillema et al., 2012; Peters et al., 2019). The validation of treatment responsive biomarkers in rare disorders, such as imaging measures of white matter integrity, will require multi-center studies using different MRI platforms at different institutions contrasting different analytic strategies to obtain reproducible and comparable assessments for volumetric and diffusion MRI. Prohl et al. address this question using traveling human phantoms across five institutions and demonstrate that inter- and intra-scanner variability were small allowing for highly reproducible assessments between and within scanners. Such studies provide crucial quality assurance methodologies as well as feasibility support for large multi-center treatment trials that utilize structural or diffusion MR imaging.

For neurodevelopmental disorders, electro- and magneto- encephalography (EEG/MEG) are non-invasive techniques with significant promise because of their ability to monitor brain activity with high temporal resolution. EEG has the advantage of being less expensive and portable for ease of clinical use. Unlike MR scanning, which requires strict head motion restriction and thus is difficult without sedation, EEG can be tolerated by many children with developmental delay. Ewen et al. discuss the criteria for validation of EEG as a biomarker in neurodevelopmental disorders, delving into both theoretical/conceptual issues as well as practical obstacles. In complement, several papers in this Research Topic provide preliminary data from a large multi-site study designed to investigate a battery of EEG and eye-tracking indices as potential biomarkers for non-syndromic autism spectrum disorder (ASD). Scientific background and the design of this study, entitled the Autism Biomarkers Consortium for Clinical Trials (ABC-CT), is described by McPartland et al.. Such multi-site trials require extreme attention to detail in order to standardize data collection across all the sites. Webb et al. detail the operating procedures and methodology that they developed in ABC-CT to address standardization and implementation issues. Finally, Levin et al. report on their findings in an investigation of the short-term test-retest reliability of EEG power spectral densities. Taken together, these three papers demonstrate excellent short-term test-retest reliability for scalp EEG profiles in children with ASD and typically developing controls once a high-degree of standardization and quality control is employed.

EEG related paradigms are being used in studies of genetically defined rare disease populations. De Stefano et al. interrogated the developmental trajectory of auditory processing in individuals with ASD and typically developing controls across the age spectrum. They presented a stimulus that entrained auditory cortex to increasing frequencies and recorded high density EEG and found disrupted gamma activity in adolescents/adults with ASD but not in children. These results suggest that certain abnormalities in neural oscillations may not emerge until later in development. Such auditory processing alterations may be helpful if they respond to treatment in older individuals but may not be as useful for earlier interventions. Importantly, the same group of investigators identified similar but more common and pronounced auditory processing abnormalities in individuals with Fragile X Syndrome (FXS) (Ethridge et al.). Furthermore, they validate their earlier findings in a different cohort of FXS participants from a new clinic and using a different EEG acquisition system and different auditory stimulus. Replicability of these EEG based biomarkers across such studies indicate that they could be scalable for use in multi-site clinical trials. Auditory processing may be abnormal not just in FXS, but also in TSC. O'Brien et al. demonstrate in a pilot study that the features of the auditory response to speech sounds, but not acoustically matched tones, can differentiate children with TSC from typically developing children. Finally, Saby et al. review the published studies of EEG and evoked potentials (auditory, visual and somatosensory) in another syndromic form of ASD and intellectual disability, Rett Syndrome. Another advantage of EEG is the possibility to connect to the cellular and molecular underpinnings through evolving developments in computational neural modeling methods. A major limitation of many of these studies is the small sample size. Larger, multi-site studies are needed to confirm the findings from these initial smaller investigations.

Aside from MRI and EEG, there are several other modalities that can utilized to investigate brain connectivity and function. These include transcranial magnetic stimulation (TMS) and eye tracking. TMS of the motor cortex can be used to measure cortical excitation and inhibition in a quantitative fashion when coupled with electromyography of the stimulated muscles (Tsuboyama et al.). Furthermore, repetitive TMS protocols in humans can be used to measure synaptic plasticity similar to long-term depression (LTD) and long-term potentiation (LTP) experimental paradigms in animal models. Jannati et al. asked whether repetitive TMS stimulation of the motor cortex could be used as a diagnostic or prognostic biomarker in children and adolescents with ASD differentiating them from age- and gender-matched typically developing controls. They also compared the developmental trajectory of LTD-like plasticity in the two groups and found differences between the ASD and TD groups. These findings argue for further investigation of TMS readout of neuronal plasticity in ASD clinical trials.

Several studies in the past have reported that individuals with ASD spend less time attending to the eyes and more time looking at mouths, bodies, and objects in comparison to typically developing controls even starting from young ages. Reisinger et al. used an emotional faces eye-tracking paradigm to ask whether they could discriminate between ASD and control groups in social attention and emotion recognition through face scanning and pupillometry. They found that the ASD group spent less time fixated on the eye region than the control group across all emotions. Pupil reactivity was also able to detect differences within the groups based on the emotional faces that were presented. However, the ASD group, like the control group, displayed increased pupil reactivity when looking at happy faces, contradicting the hypothesis that individuals with ASD process social rewards abnormally. Further studies will be needed to see whether this non-invasive modality will provide reliable and generalizable results in cohorts affected with ASD and related neurodevelopmental disorders.

One of the most important uses of biomarkers would be to stratify participants in clinical trials. With that goal, Roberts et al. performed a randomized, placebo-controlled, double-blind, single-dose study of arbaclofen (STX-209) in 25 adolescent boys with ASD. They used magnetoencephalography (MEG) to measure the response to a pure tone auditory stimulus, as well as the 40 Hz auditory steady-state response (ASSR) in the superior temporal gyrus. Their results suggested an effect of STX-209 on brain activity in only a subset (~30%) of the boys and only at a specific dose. While we are in the early stages, such studies highlight the possibility of using EEG, MEG or TMS to monitor target engagement in the brain, optimizing dosage and stratifying participants in clinical trials.

Taken together, these biomarker investigations in neurodevelopmental disorders are starting to address issues such as feasibility of acquisition, standardization, multi-site implementation, test-retest reliability, and developmental maturation. In addition to the biomarkers discussed in this Research Topic, other modalities such as actigraphy and autonomic functions are being developed and tested. It is possible that multimodal biomarker signatures that combine more than one measurement maybe more reliable and impactful. Additionally, as more data is collected and shared, advances in classification techniques with modern machine learning algorithms hold the promise to facilitate biomarker identification. Given the heterogeneity of the underlying causes of neurodevelopmental disorders and the lack of validated outcome measures that are sensitive to change to date, it is imperative that promising biomarkers are incorporated into intervention trials in this field so that their practical utility can be established. As more data are collected across age groups, genetic causes and intervention types, we are likely have a more detailed and informed perspective on the utility of biomarkers to accelerate development of therapeutics in neurodevelopmental disorders.

## Author Contributions

All authors listed have made a substantial, direct and intellectual contribution to the work, and approved it for publication.

## Conflict of Interest

MS reports grant support from Novartis, Roche, Pfizer, Biogen, Ipsen, LAM Therapeutics, Astellas, Bridgebio and Quadrant Biosciences. He has served on Scientific Advisory Boards for Sage, Roche, Celgene, Aeovian, Regenxbio and Takeda. JS is a consultant to VeraSci. The remaining author declares that the research was conducted in the absence of any commercial or financial relationships that could be construed as a potential conflict of interest.
